# Survey data of finalists and winners in the search for outstanding teachers in the Philippines, 1988–2010

**DOI:** 10.1016/j.dib.2020.106238

**Published:** 2020-08-27

**Authors:** Majah-Leah V. Ravago, Claire Dennis S. Mapa

**Affiliations:** a Department of Economics, Ateneo de Manila University, Rm 400 4/F Leong Hall, Katipunan Ave., Loyola Heights, 1108 Quezon City, Philippines; b School of Statistics, University of the Philippines, T.M. Kalaw Street, Diliman, Quezon City, Metro Manila, Philippines

**Keywords:** Teaching excellence awards, Award as signal, Impact evaluation, Financial literacy

## Abstract

The data derives from a survey of teachers who competed at the national level in the Metrobank Foundation, Inc. Search for Outstanding Teachers in the Philippines from 1988 to 2010. Conducted in March-September 2014, the survey has complete information from 252 national winners and finalists. The survey collected data on teachers’ professional profile, socio-demographic characteristics, community involvement, socioeconomic characteristic of the teachers’ household including income and expenditure, and their overall perception on the search process. It also collected information from school heads. The data collected by the survey from the school head include statistics on the educational profile of their teachers, performance indicators of the school, physical characteristics of the school, and school head's general assessment of colleagues and overall perception on the search process. The survey also includes information about the financial literacy of teachers. The dataset is in comma-separated values file (.csv) with accompanying data dictionary (.txt). The questionnaire is also included in data supplementary appendix. This data article is related to the research article, “Awards and Recognition: Do they Matter in Teachers’ Income Trajectory?” Ravago and Mapa, 2020, where data interpretation and analysis can be found.

**Specifications Table**

**Table:** 

Subject	Economics & Econometrics; and Education
Specific subject area	Impact evaluation of an award for teaching excellence; teacher's financial literacy
Type of data	Comma-separated values file (.csv) Data dictionary (.txt) Table Image Maps
How data were acquired	Face-to-face directed interview Mail survey Survey questionnaire can be accessed via Mendeley Data: Ravago, Majah-Leah; Mapa, Claire Dennis (2020), “Survey Data of Metrobank's Search for Outstanding Teachers in the Philippines, 1988–2010”, Mendeley Data, V1, doi: 10.17632/vrr2vvghx6.1
Data format	Anonymized semi-processed data in CSV format.
Parameters for data collection	The data derives from a survey of teachers who competed at the national level in the Metrobank Foundation, Inc. Search for Outstanding Teachers in the Philippines from 1988 to 2010. The survey interviewed national winners and finalists including the school heads.
Description of data collection	Conducted in March-September 2014, the survey has complete information from 252 winners and finalists. The default method of the survey was a face-to-face directed interview. Self-administered survey was conducted among 2 percent of the respondents. The conduct of this survey fulfilled the technical requirements necessary to demonstrate the use of ethical procedures in researching human participants. Implicit informed consent has been obtained from the respondents because they have agreed to be interviewed. All data gathered from the survey have been anonymized.
Data source location	The survey covered various schools in 63 provinces of the Philippines.
Data accessibility	Ravago, Majah-Leah; Mapa, Claire Dennis (2020), “Survey Data of Metrobank's Search for Outstanding Teachers in the Philippines, 1988–2010”, Mendeley Data, V1, doi: 10.17632/vrr2vvghx6.1 Anonymized data set in comma-separated values file (.csv) with accompanying data dictionary (.txt). DIB A1 Ravago and Mapa Metrobank Teachers.csv DIB A2 Ravago and Mapa Metrobank Teachers.txt DIB A3 Ravago and Mapa Metrobank School Head.csv DIB A4 Ravago and Mapa Metrobank School Head.txt Questionnaire DIB B Ravago and Mapa Questionnaire Teacher and School Head.pdf Instructions for accessing these data: Standard access via Mendeley
Related research article	Ravago, M.V., and D. Mapa, “Awards and Recognition: Do they Matter in Teachers’ Income Trajectory?” *Studies in Educational Evaluation*, Vol. 66, September 2020, 100901. https://doi.org/10.1016/j.stueduc.2020.100901

**Value of the Data**

• The data and methodology are examples of instruments in quantifying impact of awards in teaching excellence on growth of income. They can be replicated in other countries for comparison or in other discipline to further improve our understanding of the nuances of the impact of awards on income growth of recipients.
• The data is useful for school administrators who are seeking ways to reduce evaluation costs of their teacher's performance. It can be used to examine how awards can be linked to metric for promotion and rewards with pecuniary benefits.
• The data and method of collection presented here are potentially useful for other institutional award-giving body who would want to conduct impact evaluation to allow program review and improvements in their system.
• The data offers potential to scale the size of data collection to include other program of awards for teaching excellence.
• The data may be used by researchers to develop experiments and longitudinal studies that would allow estimation of dynamic effect of awards and testing for how long the effect of awards last.
• The data include financial indicators that can be used by researchers to gauge financial literacy of teachers in a developing country context. The data can potentially contribute to the improvement of required courses in strengthening the financial literacy of future teachers.
• The data can also be combined with other data on financial literacy to further evaluate the financial literacy of teachers vis-à-vis other profession.

## 1 Data Description

**Fig 1:**
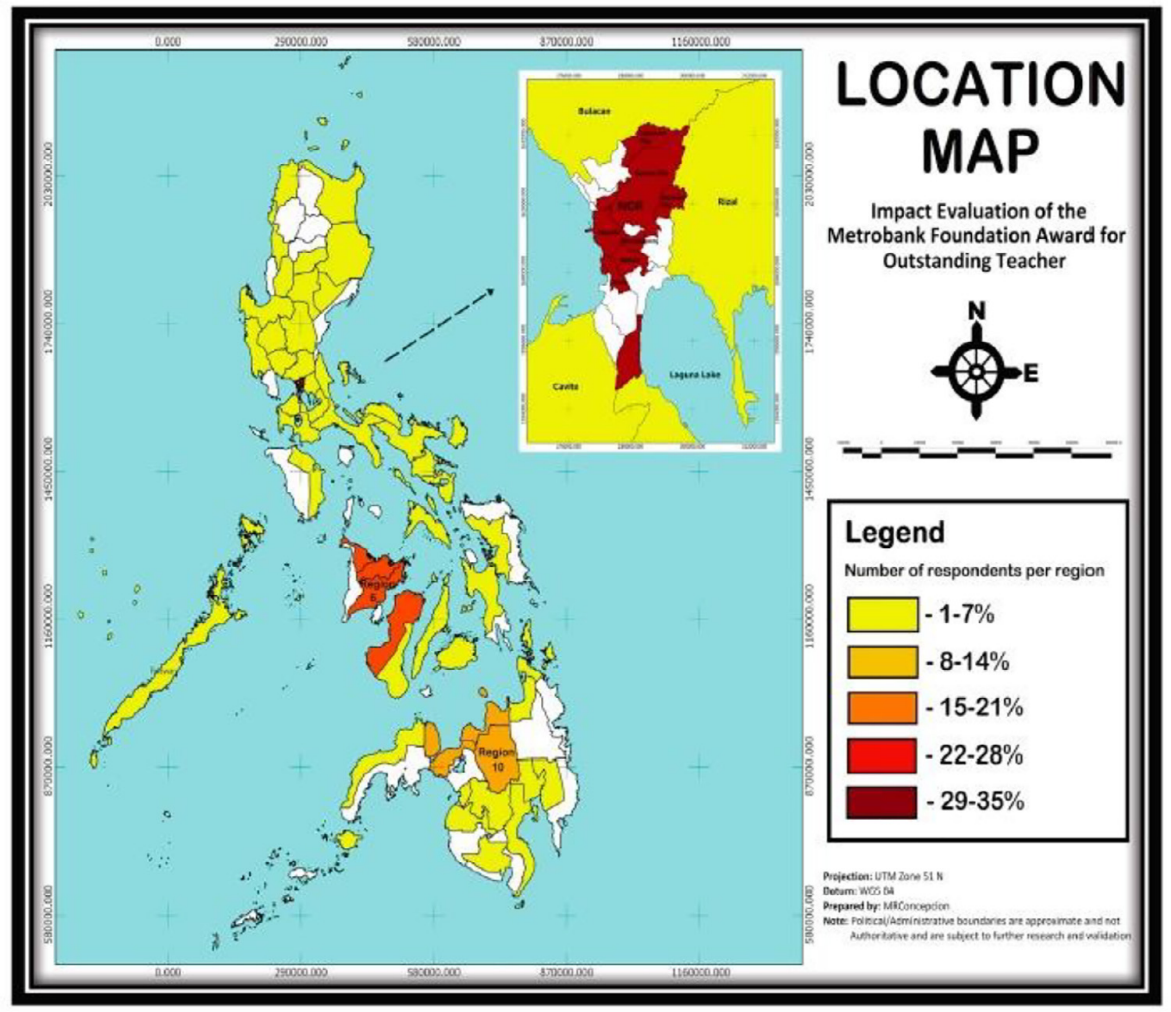
Project location map

**Fig 2:**
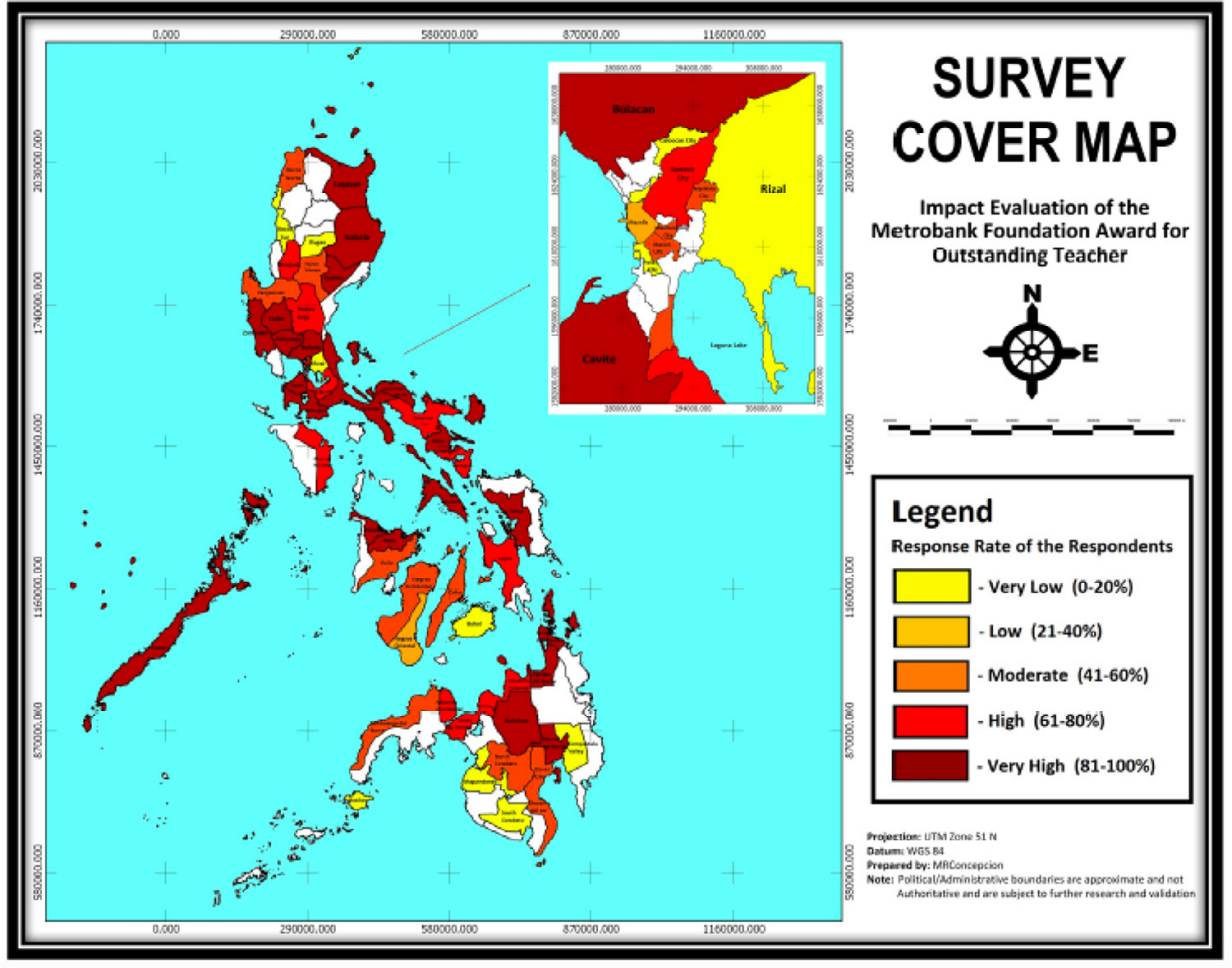
Survey cover map for all respondents

The data derives from a survey of teachers who competed at the national level in the Metrobank Foundation, Inc. Search for Outstanding Teachers in the Philippines from 1988 to 2010. Conducted in March-September 2014, the survey has complete information from 252 national winners and finalists. Supplementary Appendix A provides the anonymized data set in comma-separated values file (.csv) [1]. Fig. 1 shows the distribution of respondents across the Philippines. Fig. 2 presents the survey cover map of all respondents. The full questionnaire is provided as a Supplementary Appendix B [1]. Tables 1 and 2 give an overview of the coverage of the questionnaire for teachers and school heads, respectively. Appendix C gives the operational definitions of the specific terms used in the survey questionnaire. Table 3, Table 4, Table 5, Table 6, Table 7, Table 8, Table 9, Table 10, Table 11, Table 12, Table 13, Table 14, Table 15, Table 16, Table 17, Table 18 provide selected general results including profile of the respondents. This data article is related to the research article, Ravago and Mapa, 2020. “Awards and Recognition: Do they Matter in Teachers’ Income Trajectory?” *Studies in Educational Evaluation* [2].

**Table 1:** Coverage of the teacher questionnaire.

Block	Description/ Coverage
A	*Teacher's Profile*
	Demographic and economic characteristics of teachers. It contains data on the respondent's income (from teaching and other sources) at the time of his or her nomination and the current/ last month or last year in teaching. This also includes information about the no. of times the respondent had been nominated.
B	*Teacher's Competence*
	Information about the respondent's: teaching background; professional and community involvement; promotions and scholarships received; and other appointments, at different periods in their teaching career (e.g. from the time they started teaching, from the time of their last nomination until the survey period, before retirement, etc.).
C	*General Information on Household*
	Demographic and economic characteristics of the teacher's household members, household income, household expenditures, household assets, and type of housing.
*Rider Questions*	
D	*Financial Indicators*
	Household indicator for loan or credit, household deposit account or investment information, respondent's financial characteristics, contributions and attitudes.
E	*About Metrobank*
	Respondent's perceptions and attitudes towards *Metrobank* as an institution
F	*Teacher's Overall Perception on the Search Process*
	Perceived effects or contributions garnered by the respondent due to his/her nomination.

**Table 2:** Coverage of the school head questionnaire.

Block	Description/ Coverage
A	*School Affiliation*
	Basic information about the school, college or university; school head's knowledge about their school's awardee(s) or finalist(s) from the time of nomination; faculty's educational profile; and school head's evaluation or rating of the finalist.
B	*Questions Related to School*
	Covers general education statistics of the school, college or university, school physical characteristics, and funding sources at the time of the nominee from their school and during the survey period. This also includes the school head's perception about the applicant's direct or indirect contributions to the school.
C	*Questions Related to Fellow Teachers*
	School head's rating of his or her fellow faculty in different core characteristics of a teacher at the time of the first nominee from their school and during the survey period. This also includes the school head's perception about the applicant's direct or indirect contributions to the teachers in their school. Questions regarding other applicants from the school after the last finalist or winner, and sources of information about the search process are also in this block.
D	*Questions Related to Students*
	For primary and secondary schools, data on the mean percentage score on national achievement test per core subject and overall were obtained from the oldest available year up to the latest.
	For tertiary schools, data on the mean percentage score on licensure examination for each area of study were obtained from the oldest available year up to the latest.
*Rider Questions*	
E	*Financial Indicators*
	Household indicator for loan or credit, household deposit account or investment information, respondent's financial characteristics, contributions and attitudes.
F	*About Metrobank*
	Respondent's perceptions and attitudes towards Metrobank as an institution
G	*School Head's Overall Perception on the Search Process*
	School Head's perceived effects or contributions of the award in their school, college or university.

**Table 3:** Number of teachers by school level at the time of nomination.

Type of respondent	Primary level	Secondary level	Tertiary level	Total
Winners	65	57	46	168
(%)	(39)	(34)	(27)	(100)
Finalists	34	25	25	84
(%)	(40)	(30)	(30)	(100)
Total	99	82	71	252
(%)	(39)	(33)	(28)	(100)

Pearson Chi-square = 0.4569; p – value = 0.796

## 2 Experimental design, materials, and methods

The Metrobank Foundation's Award is the longest running and most prestigious award for outstanding teachers in the Philippines. Since its launch in 1985, more than 300 exceptional elementary, high school, and college teachers from all over the country had received this highly coveted award. Each year, there is an average of 300 nominations nationwide. Out of these nominations, the judges select 20 national finalists and finally chooses the 10 Outstanding Teachers. The awardees are typically composed of 4 elementary school teachers, 4 secondary school teachers, and 2 tertiary teachers. Section 3 of Ravago and Mapa, 2020 [2] elaborates on the Award selection process.

The *Survey of Teachers and School Heads Related to the Search for Outstanding Teachers (SOT) of the Metrobank Foundation, Inc.* collected data on the national awardees and finalists from 1988 to 2010. The respondents are primarily the 20 national finalists chosen every year from which the 10 Outstanding Teachers were drawn. The data collected is pertinent to the impact of the Award on the income growth of the teachers and to what extent it influences their success. It also includes information obtained from their respective school heads.

To some extent the collected data can also allow cursory examination of the impact of the award to the school and to the immediate community in general. The data from the survey is useful for researchers who wish to study the impact of awards on the income growth of teachers and to their professional success. In addition, the survey also collected information that can help gauge the financial literary of teachers in a developing country context.

### 2.1 Scope and coverage of the survey

The survey is designed to investigate the impact of award, measuring the actual impacts accrued by the awardees that are attributable only to the award.

The survey conducted in March to September 2014, targeted a population of national awardees and finalists from 1988 to 2010 from various provinces in the Philippines. In addition, school heads^1^ were also interviewed to gather information on the educational profile of their teachers, performance indicators of the school, physical characteristics of the school, general assessment of his or her colleagues, and overall perception of the Search and the Award.

The complete list of awardees and finalists from 1988 to 2010, obtained from the Metrobank Foundation database, was used as the reference in identifying respondents (teachers and school heads) in the survey. The conduct of this research fulfilled the technical requirements necessary to demonstrate the use of ethical procedures in researching human participants. Implicit informed consent has been obtained from the respondents because they have agreed to be interviewed. They have also been appropriately informed that answers are treated with utmost confidentiality. All data gathered from the survey have been anonymized.

### 2.2 The respondents

Guided by its objectives, the survey covered two target populations, the 380 national awardees and finalists, and the 283 school heads, from which data were separately collected. The population size was adjusted for the number of deceased and those that had been in the national finals twice.

Fig. 1 shows the national awardees and finalists’ distribution across the country: 58 percent in Luzon, 23 percent in the Visayas, and 19 percent in Mindanao. Understandably, due to proximity and relatively easy access to information about the SOT, the National Capital Region (Metro Manila) has the biggest number of national finalists, with 131 teachers. Metro Manila is followed by Region 6, with 62 national finalists.

A complete enumeration or census, wherein data is collected from the adjusted population under consideration, was employed in this survey. Due to imperfect but still statistically acceptable survey response rates^2^ for both teachers and school heads, the respondents with complete information consists of 252 teachers (about 66 percent), and 206 school heads (about 73 percent).

The survey covered 63 provinces (Fig. 2): Benguet, Ifugao, Ilocos Norte, Ilocos Sur, Pangasinan, Batanes, Cagayan, Isabela, Nueva Vizcaya, Quirino, Bulacan, Nueva Ecija, Pampanga, Tarlac, Zambales, Batangas, Cavite, Laguna, Quezon, Rizal, Oriental Mindoro, Palawan, Metro Manila, Albay, Camarines Norte, Camarines Sur, Catanduanes, Masbate, Sorsogon, Aklan, Capiz, Iloilo, Negros Occidental, Bohol, Cebu, Negros Oriental, Leyte, Samar, Zamboanga City, Zamboanga del Norte, Zamboanga Sibugay, Basilan, Bukidnon, Camiguin, Lanao del Norte, Misamis Occidental, Misamis Oriental, Davao del Norte, Davao del Sur, Compostela Valley, North Cotabato, South Cotabato, Agusan del Norte, Surigao del Norte, and Maguindanao.

### 2.3 Survey questionnaire

Two sets of survey instruments were developed: one questionnaire for teachers and another for the school heads (see Appendix B for supplementary file).

The teacher questionnaire (Table 1), consists of six blocks, namely: Block A - Teacher's profile, Block B – Teacher's competence, Block C – General information on household, Block D – Financial indicators, Block E – About Metrobank, and Block F – Respondent's overall perception of the search process. Although they are no longer part of the survey's objectives, Blocks D to F are rider questions for supplemental information regarding teachers’ financial literacy, attitudes and perception towards the search process.

The school head questionnaire (Table 2), consists of seven blocks, namely: Block A – School affiliation, Block B – Questions related to school, Block C – Questions related to fellow teachers, Block D – Questions related to students, Block E – Financial indicators, Block F – About Metrobank, and Block G – School head's overall perception on the search process. Similar to the teachers’ questionnaire, the last three blocks (E, F and G) are rider questions for supplemental information regarding the school heads’ financial literacy, attitudes and perception towards the search process.

Most of the concepts and definitions used in the survey questionnaire, follow the standard definitions used in the Philippine Statistic Authority (PSA) Family Income and Expenditure Survey [3]. The operational definitions of the terms specifically used in the survey questionnaire are given in Appendix C (included in this article).

### 2.4 Conduct of the survey

Field operations for the survey officially began in March 2014. The default method of the survey is a face-to-face directed interview. Self-administered survey was conducted among 2 percent of the respondents. A slightly adjusted version of the questionnaire was used for mailing for respondents who live in remote areas.

The respective school heads of the identified national finalists were interviewed. In cases where the principal or the dean was unavailable during the survey period or was unfamiliar with the national finalist, a recognized keyperson by the school head is interviewed.

In addition to the interviews, secondary data and personal observation of the survey enumerators are also used in gathering pertinent data from the respondents. Secondary data, which were requested in advance prior the interview schedule, include the following: teacher's curriculum vitae, service record, performance evaluation rating, school performance indicator and school mean percentage score on the national achievement test or board licensure examination.

To ensure the quality of the survey data, our team implemented measures of data quality assurance, including reporting results of the pre-test of questionnaires, training of enumerators, spot checking the fieldwork operations; sending of regular updates of fieldwork activities, including field notes, regular data dumps for initial assessment of the encoded information; final reporting on the survey; and post-survey activities from enumerators.

### 2.5 Encoding and reading the data

After the field enumeration ended in September 2014, data processing was conducted. An encoding program was developed for the survey data using MS Access to electronically capture the data from the survey. The encoding program looks exactly the same as the paper survey questionnaire to mitigate errors in encoding. The encoded data via MS Access were then exported into Microsoft Excel. Finally, data output from the different encoders were merged using the Stata software. The data is then converted as comma-separated values file (.csv) for general accessibility. Supplementary Appendix A [1] provides the data file with accompanying data dictionary (.txt).

In reading the data, when the name of the variable is alphanumeric, there is a direct correspondence in the questionnaire in most cases. For example, the variables a5_1 and a5_2 in Appendix A1 (DIB A1 Ravago and Mapa Metrobank Teachers.csv) are responses to questions A5, What is your marital status. A5.1 during your last application in SOT and A5.2 current? Otherwise, the data dictionary provides for the description of the variable. For example, the variable r_a6 is described in the data dictionary as “Recoded a6 (Active = 1, Non-active = 0)”. The correspondence among the data file, the data dictionary, and the questionnaire allows for user-friendly utilization of the data. It also reveals that most of the missing data are due to non-applicability of the question or just simply unanswered question.

### 2.6 Selected general results from the teacher's survey

From block A of the survey questionnaire for teachers [1], we can build the average profile of the teachers who competed at the national level. The total number of sample teachers with complete information is 252 teachers: 168 (66%) winners and 84 (33%) finalists. Among the 168 winners, 39, 34, and 27 percent were teaching at the primary, secondary, and tertiary levels, respectively (Table 3). Among the 84 finalists, 40, 30, and 30 percent were teaching at the primary, secondary, and tertiary levels, respectively.

Since the Metrobank Award started in the 1980s, several of the national finalists would no longer be active in the teaching profession. About 58 percent of the 252 teachers in the sample were actively teaching in 2014, at the time of the survey, while 42 percent were not. Of the 168 teachers in the winners’ group and 84 in the finalists’ group, 56 percent and 61 percent are still active, respectively. See details in Table 1 of Ravago and Mapa, 2020 [2].

The critical information in the data is the income levels of winners and finalists. Figure 1 of Ravago and Mapa, 2020 [2] shows the percentage distribution of respondents by income levels. At the time of nomination, 73 percent of the winners and 65 percent of the finalists had monthly incomes below PhP 25,000.00. In comparison, 58 percent of the winners and 63 percent of finalists had incomes between PhP 25,000.00 and PhP 50,000.00 in 2014 (1$ = PhP44 in 2014). The wide disparity of income is also reflective of the overall salary of teachers in the Philippines. Currently, the average monthly salary of public-school teachers is 72 percent higher than those in private schools (Llego, 2019 [4]). There is also a wide variation of teachers’ salaries across regions and by public and private (see more detailed discussion in Ravago and Mapa, 2020 [2]).

The responses to questions in block B [1] give information on non-pecuniary success indicators that are also critical in examining the impact of the Award. These non-pecuniary success indicators include change in educational attainment, material outputs, promotions, training, number of advisees, and community and other public service. The non-pecuniary success indicators have zero as minimum value because some national finalists joined the competition near their retirement age (see Table 2 of Ravago and Mapa, 2020 [2]).

Block B of the survey questionnaire for teachers [1] also asked about information on their teaching, appointments, and scholarships. The respondents’ status of teaching employment is related to their age. Table 4 presents their average age, both at the time of their nomination and in 2014 when the survey was conducted. The actively teaching respondents were 44 years old on average when they joined the competition, while those no longer teaching were 53 years old. In 2014, the average age of respondents, actively teaching and not teaching, was 55 and 69, respectively.

**Table 4:** Teacher's average age by status of teaching employment.

Type of respondent	Actively teaching		Not teaching	
	At the time of nomination	2014	At the time of nomination	2014
Winners	44	56	53	69
Finalists	44	53	53	69
All	44	55	53	69

Table 5 compares the respondents’ educational attainment at the time of their last nomination and in 2014. The data show vertical movements, with an increased number among those obtaining doctoral degrees. The national finalists who had bachelor's and master's degrees at the time of their nomination went on to pursue higher graduate studies. Table 6 shows the information of teachers who obtained a scholarship for their studies.

**Table 5:** Number of teachers by educational attainment.

Type of respondent	Bachelor's degree+	Master's degree++	Doctoral degree +++	Total
A. During last SOT nomination				
Winners	17	102	49	168
(%)	(10)	(61)	(29)	(100)
Finalists	10	50	24	84
(%)	(12)	(60)	(29)	(100)
B. 2014 (Time of survey)				
Winners	6	69	93	168
(%)	(4)	(41)	(55)	(100)
Finalists	1	38	45	84
(%)	(1)	(45)	(54)	(100)

+ Pearson Chi-square = 1.9853; p – value = 0.159

++ Pearson Chi-square = 0.3649; p – value = 0.546

+++ Pearson Chi-square = 1.5630; p – value = 0.211

**Table 6:** Number of teachers who studied with scholarship.

	Bachelor's degree	Master's degree	Doctoral degree	Total
A. During their nomination				
Winners	10	40	29	79
(%)	(13)	(51)	(37)	(100)
Finalists	2	16	16	34
(%)	(5)	(47)	(47)	(100)
B. 2014 (Time of survey)				
Winners	3	13	28	44
(%)	(7)	(30)	(64)	(100)
Finalists	0	2	14	16
(%)	(0)	(13)	(88)	(100)

Block C of the survey questionnaire for teachers [1] provides information on demographic and economic characteristics of the teacher's household members, household income, household expenditures, household assets, and type of housing. Table 7 gives background information of the teachers’ parents and siblings. The average family size that the teachers grew up in consists of about 8 family members (range from 2 to 16 members). The average age of teachers’ parents ranges from 55 to 75 years old; most of them are retired. The average age of the teachers’ siblings ranges from 55 to 57 years old.

**Table 7:** Profile of parents and siblings of the teacher-respondents.

	Type of respondent	
	Winners	Finalists
Family size (average)	8	8
Deceased	3	3
Age (years, average)		
Father	72	68
Mother	75	73
Siblings	57	55
Retired (count)		
Father	89	43
Mother	68	30
Siblings (average)	6	6

Table 8 provides information on the educational background of both parents and siblings. Among the winners and finalists, the father's educational attainment is evenly distributed, with about 30 percent having reached elementary, high school and college level. About 4-8 percent are PhD holders. The mother's educational attainment, on the other hand, is more skewed to those reaching elementary and high school levels only. A few had attained graduate education. About 30 percent of the winners and finalists have parents who were also teachers.

**Table 8:** Educational background of parents and siblings of teachers-respondents.

	Type of respondent		Percentage[Standard deviation]	
	Winners	Finalists	Winners	Finalists
Father				
No formal education	8	1	4.85	1.22
Elementary level	49	20	29.70	24.39
High School level	43	27	26.06	32.93
College level	48	24	29.09	29.27
Graduate studies	14	8	8.48	9.76
Vocational Course	3	2	1.82	2.44
Mother				
No formal education	6	4	3.64	4.82
Elementary level	60	27	36.36	32.53
High School level	40	28	24.24	33.73
College level	46	15	27.88	18.07
Graduate studies	8	8	4.85	9.64
Vocational Course	5	1	3.03	1.20
Across all family members (average)				
No formal education	1	1	[0.53]	[0.35]
Elementary level	1	1	[1.30]	[1.35]
High School level	1	1	[1.41]	[1.73]
College level	4	4	[2.84]	[2.08]
Graduate studies	1	1	[0.74]	[0.68]
Vocational Course	1	1	[0.57]	[0.66]

Note: Numbers for educational level may include those who had taken some years but may have not necessarily finished the degree. The residual from the total winners and finalists is due to no response.

Table 9 shows the occupational industry background of the parents of teachers. Several teachers have parents who were also in the education sector. Many parents of the national finalists were working in the agriculture, forestry and fishing sector.

**Table 9:** Occupational industry background of the teachers’ parents.

	Education	Agriculture, forestry and fishing	Others	No work	Total
Winners					
Father	18	56	83	10	168
(%)	(11)	(33)	(49)	(6)	(100)
Mother	33	18	41	75	168
(%)	(20)	(21)	(24)	(45)	(100)
Finalists					
Father	12	23	40	8	84
(%)	(14)	(14)	(48)	(10)	(100)
Mother	14	10	24	35	84
(%)	(17)	(12)	(29)	(42)	(100)

Note: “Others” is aggregate information on the occupational industry background. Education (16) and agriculture, fishery, and forestry (AFF -1) are codes following the PSA system. Residual is “no response.”

We compared the profile of the family of both the winners and finalists. Following the PSA definition, a household is defined as a social unit consisting of a person living alone or group of persons that sleeps in the same housing unit and has a common arrangement in the preparation and consumption of food. Among the 252 national finalists, only 29 percent live with multiple families in one household (Table 10). The typical family size consists of about 5 members (Table 11). This size is smaller than the family size of their first generation. On average, each family has one member attending school, working abroad, and studying abroad. In terms of educational attainment, a teachers’ family of 5 members would have, on average, two members who had finished college and two members who had obtained either a master's or doctoral degree (Table 12).

**Table 10:** Number of teachers who live with multiple families in one household.

	Yes	No	No response	Total
Winners	44	120	4	168
(%)	(26)	(71)	(2)	(100)
Finalists	29	50	5	84
(%)	(35)	(60)	(6)	(100)
Total	73	170	9	252
(%)	(29)	(67)	(4)	(100)

**Table 11:** Profile of teachers’ own households.

Average number	Type of respondent	
	Winners	Finalists
Household size	5	5
Household member currently attending school	1	1
Household member working abroad	1	1
Household member studying abroad	1	1

**Table 12:** Respondents’ average number of family members, by educational attainment

	Type of respondent	
	Winners	Finalists
Elementary level	0	0
High school level	1	1
College level	2	2
Graduate Study	2	2
Vocational course	0	0
No formal education	0	0

The survey also asked about some indicators of the quality of standards of living. These include the type of building the family resides in, type of construction materials of the building the family lives in, and information on the teachers’ family assets. On average, the teachers have been living in their current residence for about 22–25 years. A good number of winners and finalists live in a single house (Table 13). Table 14 provides additional information on the type of materials the roof is made of.

**Table 13:** Type of building/house of the respondents’ residences

	Single house	Duplex	Apartment	Condo and commercial units	Other housing units	No response	Total
Winners	131	15	5	11	4	2	168
(%)	(78)	(9)	(3)	(7)	(2)	(1)	(100)
Finalists	68	6	3	5	1	1	84
(%)	(81)	(7)	(4)	(6)	(1)	(1)	(100)
Total	199	21	8	16	5	3	252
(%)	(79)	(8)	(3)	(6)	(2)	(1)	(100)

**Table 14:** Type of construction materials for the roof of the respondents’ residences.

	Strong materials	Light materials	Mixed but predominantly strong materials	No response	Total
Winners	156	1	10	1	168
(%)	(93)	(.6)	(6)	(.6)	(100)
Finalists	75	3	4	2	84
(%)	(89)	(4)	(5)	(2)	(100)
Total	231	4	14	3	252
(%)	(92)	(2)	(6)	(1)	(100)

Table 15 and 16 provide the tenurial status of the house and lot the family resides in. About 90 percent of the national finalists owned the house and lot where their family lives. In addition, about 30 percent of the national finalists also owned a second house (Table 17).

**Table 15:** Tenure status of the land/lot occupied by the respondents’ families.

	Owned and titled	Owner - like (rights)	Rented	Rent - free with owner's permission	No response	Total
Winners	144	14	4	5	1	168
(%)	(86)	(8)	(2)	(3)	(.6)	(100)
Finalists	61	10	4	7	2	84
(%)	(73)	(12)	(5)	(8)	(2)	(100)
Total	205	24	8	12	3	252
(%)	(81)	(10)	(3)	(5)	(1)	(100)

**Table 16:** Tenure status of the housing unit occupied by the respondents’ families

	Owned	Rented	Rent - free	No response	Total
Winners	155	8	4	1	168
(%)	(92)	(5)	(2)	(.6)	(100)
Finalists	72	5	5	2	84
(%)	(86)	(6)	(6)	(2)	(100)
Total	227	13	9	3	252
(%)	(90)	(5)	(4)	(1)	(100)

**Table 17:** Respondents’ ownership of another housing unit

	Yes	No	No response	Total
Winners	53	114	1	168
(%)	(32)	(68)	(.6)	(100)
Finalists	32	50	2	84
(%)	(38)	(60)	(2.)	(100)
Total	85	164	3	252
(%)	(34)	(65)	(1)	(100)

Information on the presence or absence of various assets was also obtained to also indicate the respondents’ standard of living. Table 18 shows that vehicles, appliances, and gadgets are the most common assets owned by both winners and finalists.

**Table 18:** Assets owned by teachers.

	During last SOT nomination		2014	
Number	Winners	Finalists	Winners	Finalists
Housing unit	133	63	149	72
(%)	(79)	(75)	(89)	(86)
Land	139	62	147	70
(%)	(83)	(74)	(88)	(83)
Mechanized farm equipment	5	2	8	4
(%)	(3)	(2)	(5)	(5)
Livestock and poultry	20	11	26	16
(%)	(12)	(13)	(15)	(19)
Vehicles	86	44	114	60
(%)	(51)	(52)	(68)	(71)
Appliance and gadgets	158	80	160	82
(%)	(94)	(95)	(95)	(98)
Boats	2	0	1	1
(%)	(1)	(0)	(.6)	(1)
Jewelries	75	42	84	45
(%)	(45)	(50)	(50)	(54)

**Table 19:** Teacher's household availment of loan or credit.

		Yes	No	Total
Active	Winner	66	28	94
		(70.21)	(29.79)	(56.29)
	Finalist	31	18	49
		(63.27)	(36.73)	(59.76)
Non - active	Winner	16	57	73
		(21.92)	(78.08)	(43.71)
	Finalist	11	22	33
		(33.33)	(66.67)	(40.24)
Total	Winner	82	85	167
		(49.10)	(50.90)	(67.07)
	Finalist	42	40	82
		(51.22)	(48.78)	(32.93)
	Overall	124	125	249
		(49.80)	(50.20)	(100.00)

Note: Number in parenthesis are percentages.

**Table 20:** Average number of accounts of teachers.

	Active		Non - active	
	Winner	Finalist	Winner	Finalist
Savings account	2.33	2.35	2.70	2.00
Current account	1.09	1.07	1.32	1.73
Time deposit	1.76	0.85	1.78	1.00
Savings certificate	0.92	0.00	0.60	1.00
Bond	1.45	0.25	1.40	1.00
Mutual fund	1.38	0.00	1.11	1.50
Others	1.33	1.14	1.33	1.00

**Table 21:** Type of accounts where teachers are likely to put their surplus money.

	Winner	Finalist	Frequency (n=247)	Percent
Deposit/Save on Bank	104	55	159	64.37
Investments (Stocks, Mutual Funds, UITFS)	42	23	65	26.32
Put up/ Invest in Business/Buy goods for sale/inventory	23	13	36	14.57
Keep in piggy bank for emergency	16	4	20	8.10
Pay debt	20	11	31	12.55
For tuition	19	11	30	12.15
Life insurance/Pension plan	22	12	34	13.77
Educational plan	14	9	23	9.31
Buy car/appliances	14	7	21	8.50
Buy house/condo	18	8	26	10.53
Buy land	22	17	39	15.79
Renovate/house improvements	30	16	46	18.62
Vacation travel local/abroad	90	43	133	53.85
Shopping	32	20	52	21.05
Help parents/relatives	57	29	86	34.82
Give to charity/church	98	40	138	55.87
Others	25	10	35	14.17

Others includes for financial assistance to students and scholars, recreation (books, arts and paintings), benefits for house helpers, etc.

### 2.7 Selected results from rider questions

Block D - F of the survey questionnaire for teachers [1] are rider questions. Block D gives important indicators that are useful in gauging financial literacy of teachers in a developing country context. These indicators include availments of loan or credit, household deposit account or investment information, respondent's financial characteristics, contributions and attitudes.

Block D and F of the survey questionnaire for teachers and school heads [1] are questions pertaining to Metrobank and the respondent's perception on the search process and the award. Following are some of the responses.

From the finalists and winners:

• “It has boosted my morale because it has enhanced my value as a teacher. It significantly influenced my promotion and my employment in Brunei was because of Metrobank. My current economic status is triggered by Metrobank. I also became very confident about myself. It opened many opportunities. I am more empowered as a teacher and to give impact to students. Once you tell them that you are an awardee, hats off. You can meaningfully relate to the students. Dignity, credibility, personal aura, it's all there, they treated you respectfully.”
• “It made me believe that I can do something more, a group look up to me on what I do. It really made me believed in myself. It also became a challenge and inspiration, the process itself. Also a good tribute to the retirees.”
• “Winning Metrobank Outstanding Teacher is life changing. It brought significant personal development. It really made a difference to my family, community, and professional growth. Where I am now, that is because of Metrobank.”
• “Wish came true! I was very lucky to win the award. After winning the award, there were many opportunities that opened to me and my family. It was my golden year. I experienced several things that I never expected will happen to me. I am very blessed to be a Metrobank awardee.”
• “Metrobank changed my life.”
• “It improved my economic status. I used the prize money as capital.”
• “I gave P25,000.00 to my school to buy chairs and tables for the kids. I became more helpful especially to those who are in need. I strengthened my teaching skills so that Metrobank will not say that they made a mistake in awarding me. Because of it I also tried other areas in teaching.”
• “There were lots of changes. First, I was promoted and became a model of the community. It served as my stepping stone for becoming a principal. I just keep convincing the teachers to join the search. In fact, I became the marketing agent for Metrobank SOT and served as a philanthropic corporate social responsibility of recognizing the outstanding teachers.”
• “It is the best experience in my teaching career. It is a realization of my dedication and efficiency as a teacher. I am proud to have this “best” award that others don't have. It is a legacy for my family, school, community, and country.”
• “Tremendous! It improved the system in places I operate.”
• “I established excellence, more often I am invited as speaker. Metrobank is an ideal people developer.”
• “It contributed in terms of financial aspect. In terms of accreditation, I have a certificate to show. Also, I can encourage others to join and joining the SOT resulted to my career advancements. I suggest that they (Metrobank) should visit schools to encourage teachers to apply and to promote the SOT. Also, increase the prize money for finalists, there's a big gap in the prize money for winner and finalist. I think the judges are good but request too many documents.”
• “It is good to receive recognition but even without it one should still be excellent in their work all the time. After the finals, finalists no longer had any involvement with Metrobank. NOTED, for example, is only for national winners and they are involved in long – term. I hope finalists can also be involved. In addition, involve teachers in the Top 20, especially those in the provincial level. They should be given due recognition kasi they are usually those who teach 100%.”
• “It made me realized that it was difficult more than my thesis dissertation.”
• “It didn't add too much to my career because I already achieved a lot.”

From the school head:

• “Transformation! The school before was labeled as dying school because of lowering number of enrollees. After having a finalist of Metrobank, it regains the trust of the community. Thereof, there was an increase in enrollment, revived the participation of community in school activities. Lastly, the school transformed from dying school to well performing school.”
• “The search for outstanding teacher puts the school into global level; bring prestige and dignity in school.”
• “The laboratory equipment of the school was increased and the things are more organized. The teachers were inspired to do their best and the students rejoiced with the award.”
• “It had good effect on the part of the school. The enrollment increased during the following years. It also improves the competencies of the teachers because of the computer training shared by the awardee. It uplifted the name of the school in the community due to the prestige of the award.”
• “It gave an inspiration and motivation among the teachers. Our school also became known in the province.”
• “There is no impact. The increase in enrollment rates in the university can be attributed to the university's reputation and not the award. I suggest that the funds given to the university from the prize money should be provided continuously for a given number of years to truly create impact. There should be concrete projects.”

## Adherence to Ethical Requirements

The authors certify that the conduct of this research has fulfilled the technical requirements necessary to demonstrate the use of ethical procedures in researching human participants. Implicit informed consent has been obtained from the participants because they have agreed to be interviewed. They have also been appropriately informed that answers are treated with utmost confidentiality. All data gathered from the survey have been anonymized.

## CRediT author statement

MVRavago conceptualized the project, collaborated with the second author in the design and creation of the survey; supervised the data collection process; wrote, reviewed, and revised the manuscript.

DSMapa collaborated with the first author in the design and creation of the survey; was involved in data collection process; and supervised the data encoding process.

Appendix. Supplementary materials

Research data for this article

## Declaration of Competing Interest

The authors declare that they have no known competing financial interests or personal relationships which have, or could be perceived to have, influenced the work reported in this article.

## Appendix Supplementary materials

Appendix A. Anonymized data set in comma-separated values file (.csv) with accompanying data dictionary (.txt). DIB A1 Ravago and Mapa Metrobank Teachers.csv DIB A2 Ravago and Mapa Metrobank Teachers.txt DIB A3 Ravago and Mapa Metrobank School Head.csv DIB A4 Ravago and Mapa Metrobank School Head.txt Image, application 1

Appendix B. Supplementary file \elsamp #x2013; Questionnaire DIB B Ravago and Mapa Questionnaire Teacher and School Head.pdf Image, application 2

Appendix C. Operational definitions of the specific terms used in the survey questionnaire (with this article) Image, application 3

## Footnotes

1 This survey collected data from the heads of schools, colleges or universities where the national awardees and finalists from 1988 to 2010 teach / taught in during the selection process. Some schools, colleges or universities have multiple awardees / finalists.

2 This measures the percentage of respondents in the survey, who were successfully interviewed and provided complete information.
