# Supplementary material for: Survey data of finalists and winners in the search for outstanding teachers in the Philippines, 1988–2010
Source: Data Brief. 2020 Aug 27;32:106238. doi: 10.1016/j.dib.2020.106238 (PMC7474403; doi:10.1016/j.dib.2020.106238)
Supplement: Supplementary file 2 — Appendix B. Supplementary file \elsamp #x2013; Questionnaire DIB B Ravago and Mapa Questionnaire Teacher and School Head.pdf [file mmc2.pdf]

SOT Survey Questionnaire I
(For the teacher only)
Strictly Confidential

Control No.: A T - ___

Appendix B: Supplementary file - Questionnaire
Survey Data of Finalists in the Metrobank Foundation Search for Outstanding Teachers 1988-2010

Majah-Leah V. Ravago[1]**, Claire Dennis S. Mapa**[2]
1. Ateneo de Manila University 2. University of the Philippines

ENUMERATOR'S INFORMATION
Name of Enumerator: ____________________

Date:
Time Started:
Time Finished:

Note for the Enumerator:
____
____

For your Supervisor only

Year of the respondent's latest application for SOT ……………………………
Respondent: 1 - Finalist 2 - Winner
School level that the respondent belongs
1 - Primary 2 - Secondary 3 - Tertiary

SOT - Search for Outstanding Teachers, Metrobank Foundation, Inc.

A. RESPONDENT'S PROFILE

(A1) Name of Respondent:
(A1.1) Surname (A1.2) Given Name (A1.3) Middle Name (A1.4) Ext.(e.g. Sr, Jr)

(A2) Home Address:
(A2.1) Barangay (A2.2) Municipality/City (A2.3) Province (A2.4) Region

(A3) Contact Information
Mobile Number: Residence Tel. No.: Personal Email Address:

(A4) Birth Place: ____

(A5) What is your marital status ……….
(A5.1) during your last application in SOT? ☐ (A5.2) Current? ☐

Codes for Marital Status

1 - Single	3 - Widow/Widower
2 - Married	4 - Separated

(A6) Did you retire or resign from teaching? (1 - Retire, 2 - Resign, 3 - No) ☐
If No, proceed to A10 and A11 but skip A10.3 and A11.3 respectively ….

(A7) If your answer in A6 1 or 2, in what year did you retire/resign? ☐

(A8) After retiring/resigning, what is your **main occupation**? Refer to codes below. ☐

Codes for Occupation

121 - Director and Chief Executive of Corporations	215 - Engineers and other related profession	621 - Livestock and Dairy Farmer
131 - General Manager or Managing Proprie	221 - Life Science Professional	622 - Poultry Farmer
141 - School Supervisors and Principal	241 - Business Professional	641 - Aqua Farm Cultivator
142 - Transport and Communication Service Supervisor	244 - Social and related science professionals	642 - Inland and Coastal Water Fisherman
144 - Sales Supervisor in Wholesale and Retail Trade	245 - Writer and creative or Performing Artist	711 - Miner, Shotfirer, Stone Cutter and Carver
211 - Physicist, Chemist, and related professionals	611 - Field Crop Farmer	999 - None
212 - Mathematician, Statistician, and other related professionals	612 - Orchard Farmer	99 - Others, please specify ______________________

(A8.1) What type of industry? Refer to codes below. ☐

Codes for Industry

1 - Agriculture, Forestry, and Fishing	12 - Real Estate Activities
2 - Mining and Quarrying	13 - Professional, Scientific and Technical Services
3 - Manufacturing	14 - Administrative and Support Service Activities
4 - Electricity, Gas, Steam, and Air-conditioning Supply	15 - Public Administrative and Defence, Compulsory Social Security
5 - Water Supply, Sewerage, Waste Management, and Remediation Activities	16 - Educatio
6 - Construction	17 - Human Health and Social Work Activities
7 - Wholesale and Retail Trade, Repair of Motor Vehicles and Motorcycles	18 - Arts, Entertainment, and Recreation
8 - Transportation and Storage	19 - Activities of Private Households as Employers and undifferential goods, and services and producing activities of household for own use
9 - Accommodation and Food Services Activities	20 - Activities of Extraterritorial Organizations and Bodies
10 - Information and Communication	
11 - Financial and Insurance Activitie	

(A9) How many years are you in your occupation in A8? ☐

(A10) What is your estimated **individual monthly income** from teaching………. **if retired skip A10.2, otherwise skip A10.3**
(A10.1) during your last application in SOT? ☐ (A10.2) current/last month? ☐ (A10.3) last year in teaching, if retire/resign? ☐

(A11) Do you have any other source of income in addition to your income from teaching ….. (1 - Yes, 0 - No)
(e.g. consulting, business, lending operations, commission from sales etc.)
If retired skip A11.2, otherwise skip A11.3
(A11.1) during your last application in SOT? ☐ If Yes, what type of industry? ☐
(A11.2) current? ☐ If Yes, what type of industry? ☐
(A11.3) from your last year in teaching? ☐ If Yes, what type of industry? ☐
Refer to codes in A8.1

(A12) Consider your income and economic circumstances compared with all the people in your school.
Where would you place yourself in terms of your present economic circumstances? ☐
If retired, consider your last paid position.

1 - Top 1% - 5%	3 - Top 11% - 20%	5 - Lowest 50%
2 - Top 6% - 10%	4 - Top 21% - 50%	

(A13) How many times did you apply for the Metrobank's Search for Outstanding Teacher Award? ☐

B. RESPONDENT'S PROFESSIONAL PROFILE

I. Teaching

(B1) What is your highest educational attainment ……………	(B1.1) during your last SOT application	(B1.2) current/last year
1. Educational Level		
2. Field of Specialization		
3. Scholarship/s*, if any (1 - Yes, 2 - No)		

Note: Scholarship/s only referring to the highest degree on B1.1 and B1

Codes for Educational Lev
1 - Bachelor's Degre 2 - Masteral Degree 3 - Doctoral Degree

Codes for Field of Specialization

1 - Early Childhood Education	8 - General Science	15 - Mathematics	22 - Islamic Studies
2 - Special Education	9 - Biology	16 - Physics	23 - Music and Arts
3 - Pre-school Education	10 - Chemistry	17 - Economics	24 - Library & Information Science
4 - Physical Education	11 - English	18 - Statistics	25 - Speech and Theater Arts
5 - General Education	12 - Filipino	19 - Business and Accounting	26 - Literature
6 - Industrial Education	13 - History	20 - Engineering	99 - Others, please specify
7 - Values Education	14 - Home Economics	21 - Social Science	______________________

If retired skip B2.2, otherwise skip B2.3

(B2) What is your _____ load (in number of hours per week)	(B2.1) during your last SOT application?	(B2.2) current/last year?	(B2.3) from your last year in teaching? (If retire/resign)
1. Teaching*			
2. Committee			
3. Administrative Work			
4. Research			
5. Others, please specify			
______________________________			
6. Teaching Load in other School/s, if any			
______________________________			

Note: Put N/A if not applicable
If teaching load exceeds 40 hours, seek clarification

(B3) Number of Advisees in ………. **(FOR COLLEGE ONLY)**

(B3.1) Undergraduate	
(B3.2) Masters	
(B3.3) Ph.D.	

(B4) How many material outputs did you produce related to teaching ……..	(B4.1) from the time you started teaching* until now?	(B4.2) from the time of your last SOT application until now?
1. Instructional Materials		
1.1 Textbooks	___________	___________
1.2 Reference Materials	___________	___________
1.3 Manuals	___________	___________
1.4 Workbooks	___________	___________
1.5 Modules	___________	___________
1.6 Evaluation Instruments	___________	___________
1.7 I.T. Materials	___________	___________
1.8 Others, please specify: _______________	___________	___________
2. Published Research		
2.1 Books/Book Chapters	___________	___________
2.2 Journals Articles	___________	___________
2.3 Non - journal Articles	___________	___________
2.4 Published Reports	___________	___________
2.5 Others, please specify: _______________	___________	___________
3. Original Creative Outputs		
3.1 Song Compositions	___________	___________
3.2 Poems	___________	___________
3.3 Artworks and Paintings	___________	___________
3.4 Films	___________	___________
3.5 Musical or Stage Plays	___________	___________
3.5 Others, please specify: _______________	___________	___________

Note: Cumulative number of material outputs from the time the respondent started teachin

II. Professional and Community Involvement

(B5) Have you been involved in professional or community activities outside of teaching from the time you started teaching? (1 - Yes, 0 - No) ☐

If Yes, go to B5.1 otherwise proceed to B6.

(B5.1) Number of involvement in professional or community activities outside of teaching did you attend? Activities such as…	from the time you started teaching?		
	(B5.11) Local*	(B5.12) National	(B5.13) International
1. Training programs *(e.g., organizer, consultant, speaker, etc.)*			
2. Participation in professional organizations *(e.g. Teachers' Association)*			
3. Participation in community and civic movements *(e.g. Homeowners' Association, Red-Cross)*			
4. Consulting Works *(e.g. Commission Works)*			
5. Others, please specify ______________________			

***Local covers Barangay, City/Municipality, Province and Region**

(B6) Did you win any professional or community service awards from the time you started teaching? (1 - Yes, 0 - No) ☐

(e.g. Gawad Guro, Palanca Awards for Literature, Ulirang Ina or Ama, Model Family Award, etc.)

If YES, go to B6.1, otherwise proceed to B7

No.	(B6.1) Awards received from the time you started teaching			
	(B6.11) Year	(B6.12) Name of Award	(B6.13) Type of Award	(B6.14) Any cash grants? If Yes, specify the amount.
1				
2				
3				
4				
5				
6				
7				
8				
9				
10				

Note: Use additional sheet if necessary

Codes for Type of Award
1 - Local (Municipal, Provincial, Regional)
2 - National
3 - International

III. Promotions and Scholarships

(B7) Did you have any promotions from the time you started teaching? (1 - Yes, 0 - No)

If YES, got to B7.1, otherwise proceed to B7.2

Please list down your promotions

No.	(B7.1) Promotions from the time you started teaching.	
	(B7.11) Year	(B7.12) Position/Rank
1		
2		
3		
4		
5		
6		
7		
8		
9		
10		

Note: Use additional sheet if necessary

(B7.2) If NO, what were the reasons why you were not promoted?

____

____

____

(B8) Did you attend any trainings including post-doctoral studies from the time you started teaching? (1 - Yes, 0 - No)

If YES, fill - up the table below, otherwise proceed to B9

List down all the trainings attended including post-doctoral studies

No.	(B8.1) Year	(B8.2) Name of Training	(B8.3) Type of Training (Refer to codes below)	(B8.4) Term of Training (Refer to codes below)	(B8.5) Source of Fund (e.g. scholarship, own expences). Please specify.	(B8.6) Estimated Value	(B8.7) Is the training coursed through Metrobank Foundation? (1 - Yes, 0 - No)
1							
2							
3							
4							
5							
6							
7							
8							
9							
10							

Note: Use additional sheet if necessary

Codes for Type of Training
1 - Local (Municipal, Provincial, Regional)
2 - National
3 - International

Codes for Term of Training
1 - Short Term (1 day to 3 weeks)
2 - Long Term (Greater than 3 weeks)

IV. Other Appointments

(B9) Did you have any position in any government office, private company or NGO? (1 - Yes, 0 - No) ☐

If Yes, fill - up the table below otherwise proceed to B10

List down all positions held from after teaching.

Agency	(B9.1) Year	(B9.2) Name of Agency	(B9.3) Position	(B9.4) Number of Years
1. Government Office				
2. Private Company (including family owned business/company)				
3. Non-government Organization				

Note: Position in B9 is NOT political position

(B10) Did you run for any government position? (1 - Yes, 0 - No) ☐

If YES, fill - up the table below otherwise proceed to Part C

List down all political positions run for

No.	(B10.1) Year	(B10.2) Position*	(B10.3) Is it for local or national position? (1 - local, 2 -nat'l)	(B10.4) Did you win the election? (1 - Yes, 0 - No)	(B10.5) If Yes, how many years did you render your service?
1					
2					
3					
4					
5					

Note: Political Positions
Use additional sheet if necessary

C. GENERAL INFORMATION ON HOUSEHOLD

I. Demographic and Economic Characteristics of Household Members

(C1) We will now ask you about your family background

No.	(C1.1) Name	(C1.2) Highest Educational Attainment	(C1.3) Is [Name] deceased?	(C1.4) Age as of last birthday	(C1.5) Did [NAME] retire?	If C1.3/C1.5 is YES, what is [NAME]'s ….		(C1.8) Agency/ Organization/ Company	(C1.9 [Mu
			1 - Yes<br>0 - No		1 - Yes<br>0 - No	(C1.6) Primary Occupation	(C1.7) Primary Industry		1st
1									
2									
Siblings**									
3									
4									
5									
6									
7									
8									
9									
10									
11									
12									

****In descending order, starts with the eldest down to the youngest**
Use additional sheet if necessary

Codes for Highest Educational Attainment
1 - Elementary Level
2 - Elementary Graduate
3 - High School Level
4 - High School Graduate
5 - College Level
6 - College Graduate
7 - Master's or units earned
8 - Ph.D. or units earned
9 - Vocational Course
10 - No Formal Education

Codes for Occupation
121 - Director and Chief Executive of Corporations
131 - General Manager or Managing Proprietor
141 - School Supervisors and Principal
142 - Transport and Communication Service Supervisor
144 - Sales Supervisor in Wholesale and Retail Trade
211 - Physicist, Chemist, and related professionals
212 - Mathematician, Statistician, and other related professionals
215 - Engineers and other related professionals
221 - Life Science Professional
241 - Business Professional
244 - Social and related science professionals
245 - Writer and creative or Performing Artist
611 - Field Crop Farmer
612 - Orchard Farmer
621 - Livestock and Dairy Farmer
622 - Poultry Farmer
641 - Aqua Farm Cultivator
642 - Inland and Coastal Water Fisherman
711 - Miner, Shotfirer, Stone Cutter and Carver
999 - None
99 - Others, please specify
____

Codes for Industry
1 - Agriculture, Forestry, and Fishing
2 - Mining and Quarrying
3 - Manufacturing
4 - Electricity, Gas, Steam, and Air-conditioning Supply
5 - Water Supply, Sewerage, Waste Management, and Remediation Activities
6 - Construction
7 - Wholesale and Retail Trade, Repair of Motor Vehicles and Motorcycles
8 - Transportation and Storage
9 - Accommodation and Food Services Activities
10 - Information and Communication
11 - Financial and Insurance Activities
12 - Real Estate Activities
13 - Professional, Scientific and Technical Services
14 - Administrative and Support Service Activities
15 - Public Administrative and Defence, Compulsory Social Security
16 - Education
17 - Human Health and Social Work Activities
18 - Arts, Entertainment, and Recreation
19 - Activities of Private Households as Employers and undifferential goods, and services and producing activities of household for own use
20 - Activities of Extraterritorial Organizations and Bodies

Codes for Agency/Organization/Company
1 - Government Agency
2 - Private Company
3 - Non-Government Organization

Codes for Political Positions
1 - Brgy. Councillor
2 - Brgy. Captain
3 - Municipal Councillor
4 - Mayor
5 - Vice Mayor
6 - Board Member
7 - Vice Governor
8 - Governor
9 - Congressman/Congresswoman
99 - Others, please specify
____

Codes for Religion
1 - Roman Catholic
2 - Muslim
3 - Non - Catholic/Non - Muslim
(e.g. Saksi, INC, Methodist, Rizalist, C
99 - Others, please specify
____

(C3) Respondent's Household** Profile

HH Member No.	All Persons<br>(C3.1) Name of household member beginning with the household head INCLUDING YOURSELF	(C3.2) Relationship to HH head	(C3.3) Sex<br>1 - M<br>2 - F	(C3.4) Age as of last birth-day	(C3.5) Religion (Refer to codes in C1)	(C3.6) Ethnicity (Refer to codes in C1)
1						
2						
3						
4						
5						
6						
7						
8						
9						
10						

Note: Use another sheet if necessary

****NSO Definition:** A **household** is a social unit consisting of a person living alone or a group of persons who sleep in the same housing unit and ha common arrangement in the preparation and consumption of food

Codes for Relationship to HH head
1 - Head
2 - Wife/Spous
3 - Son/Daughter
4 - Brother/Sister
5 - Son-in-law/Daughter-in-lav
6 - Grandson/Granddaughter
7 - Father/Mother
9 - House Helper
99 - Other relative, please speci
____

Codes for Marital Status
1 - Single
2 - Married
3 - Widow/Widower
4 - Separate

HH Member No.	Name of household member beginning with the household head **INCLUDING YOURSELF**	5-24 Years Old<br>(C3.7) Is [NAME] currently attending school? (1 - Yes, 0 - No)	15 Years Old & Over<br>(C3.8) Overseas Filipino Indicator (Refer to codes below)	5 Years Old & Over<br>(C3.9) Marital Status	(C3.10) What is the highest educational attainment of [NAME]? (Refer to codes in C1)
1					
2					
3					
4					
5					
6					
7					
8					
9					
10					

Note: Use another sheet if necessary

Codes for Overseas Filipino Indicator
1 - Worker abroa
2 - Student abroad
99 - Others, please specify
____

II. Household Income

HH Member No.	Name of household member beginning with the household head **INCLUDING YOURSELF**	Permanent/Regular: (C4) During the **LAST 12 MONTHS**, what is the **average monthly income** from permanent/regular employment?		Seasonal/Occasional: (C5) During the **LAST 12 MONTHS**, what is the **average monthly income** from seasonal/occasional employment? (e.g. sidelines, consultancy works)	
		(C4.1) Cash	(C4.2) Kind	(C5.1) Cash	(C5.2) Kind
1					
2					
3					
4					
5					
6					
7					
8					
9					
10					

Note: In cash includes allowances, honoraria, bonus, commissions and others while in kind includes housing, food, grocery, clothing, medical benefits, etc.

(C6) Other Sources of Income (not listed in C4 - C5)	(C6.1) During the **LAST 12 MONTHS**, did you or any member of your family receive income from other sources? (1 - Yes, 0 - No)	(C6.2) **Monthly Average**	(C6.3) If YES, how often these remittances are received? (Refer to codes below)
1. Remittances from family members/relatives working abroad temporarily (not more than 5 straight years			
2. Cash receipts, support, assistance and relief from domestic sources			
3. Rentals received from non-agricultural lands, buildings, spaces, and other properties			
4. Interests from bank deposits and loans extended to other families			
5. Pension and retirement, workmen's compensation and social security			
6. Dividends from investments (stocks, bonds, etc.)			
7. Other sources of income NEC (not elsewhere classified), please specify			

Codes for frequency of Remittance

1 - Weekly 2 - Bi-monthly 3 - Monthly 4 - Quarterly 5 - Semi-annually 6 - Annually 7 - Occasionally 8 - Only once

III. Expenditures

During the last 12 months, how much on the average is your household ***WEEKLY*** *consumption of the following? (Include all food items consumed from purchases made whether in cash or on credit, received as gifts, and own-produced, Round off to the nearest peso.*

(C7) Food consumed at home ___,___

(C8) Food regularly consumed outside the home (e.g., meals at schools, places of work, restaurants, merienda, or snacks, etc.) ___,___

(C9) Alcoholic beverages (beer, tuba, basi, lambanog, brandy, whisky, rhum, etc.) ___,___

(C10) Tobacco (cigarettes, cigar, betel nut, chewing tobacco, teal tobacco, etc.) ___,___

During the last 12 months, how much on the average is your household ***MONTHLY*** *expenses/consumption on the following? (Include all expenses/consumption whether purchased/paid in cash/credit, received as gifts or own-produced. Round off to the nearest peso.)*

(C11) Fuel, light, and water (charcoal, firewood, LPG, kerosene/gas, electricity, candle, oils, water, etc.) ___,___

(C12) Transportation and communication (bus, jeepney, tricycle, transport fare, water transport fare, gasoline/diesel, driver's salary, telephone bills, postage stamps, telegrams, driving lesson fees, feeds for animals used for transport, etc.) ___,___

(C13) Household operations (laundry soap and detergent, starch, floor wax, insect spray/rat and mosquito killer/coil, cleanser/scouring pad, air freshener/deodorizer, fluorescent/ incandescent bulbs, matches, brooms, husks, battery, etc.) ___,___

(C14) Personal care and effects (cleansing cream, body deodorant, lotion, baby oil, toilet/bath soap, tissue paper, toothpaste, sanitary napkin, shampoo, jewellery, handbag, wallet, wristwatch, haircut, manicure/pedicure, etc.) ___,___

During the last 12 months, how much on the average is your household ***ANNUAL*** *disbursements/expenditures on the following? (Include expenditures whether purchased/paid for in cash or on credit, received as gifts. Round to the nearest peso).*

(C15) Clothing, footwear and other wear (clothing & ready-made apparel, footwear, sewing materials, accessories, service fees, etc.) ___,___

(C16) Education (tuition fees, graduation fees, allowance for family member studying away from home, books, school/supplies, etc.) ___,___

(C17) Recreation (children bicycle & playcars, dolls, balls mahjong sets, admission tickets to movies, rental of video tapes, food for pets, etc.) ___,___

(C18) Medical care (drugs & medicines, hospital room charges, medical and dental charges, other medical goods & supplies, herbal medicines, etc.) ___,___

(C19) Non-durable furnishing (dinnerware, glassware, silverware, plastic ware, kitchen utensils/knives, mosquito net, pillow, pillow cases, etc.) ___,___

(C20) Durable furnishing (refrigerator, cooking range/stove, washing machine, T.V, cassette recorder, electric fan, etc.) ___,___

(C21) Taxes (income tax, real estate tax, car registration, toll fees & other license, residence certificate, etc.) ___,___

(C22) Health and Life Insurances ___,___

(C23) House maintenance and repair (carpentry materials, electrical materials, masonry paint, plumbing materials, etc.) ___,___

(C24) Special occasions (birthday, wedding, baptismal, anniversary, family reunion, etc.) ___,___

(C25) Gifts and contributions to others (gifts & assistance to private individuals outside the family, contribution to church, donations, etc.) ___,___

(C26) Other disbursements (purchase/amortization of real property and/or vehicles; payments of cash loan; instalments for appliances; loans granted, etc.) ___,___

IV. Household Assets

(C27) Assets		(C27.1) Do you own the following assets? (1 - Yes, 0 - No)				(C27.2) Curr (Refers to the ansv (If the HH owns more th get the s
		(C27.11) During SOT Application	(C27.111) If YES, how many?	(C27.12) Current**	(C27.121) If YES, how many?	
1. Houses						
2. Land	2.1 Farmland					
	2.2 Residential					
	2.3 Commercial					
3. Mechanized Farm Equipment	3.1 Tractor					
	3.2 Thresher					
	3.3 Weeder					
	3.4 Others, please specify ______________________					
4. Livestock and Poultry	4.1 Carabao					
	4.2 Cow					
	4.3 Hogs					
	4.4 Goat					
	4.5 Chicken					
	4.6 Ducks					
	4.7 Others, please specify ______________________					
5. Vehicles	5.1 Trucks for Hire					
	5.2 Bus for Hire					
	5.3 Jeepney for Hire					
	5.4 Jeepney for personal use					
	5.5 Tricycle for Hire					
	5.6 Tricycle for personal use					
	5.7 Car for Hire (e.g. Taxi)					
	5.8 Car for personal use					
	5.9 Motorcycle for personal use					
	5.10 Bicycle					
	5.11 Others, please specify ______________________					
6. Appliances and Gadgets	6.1 Radio					
	6.2 Washing machine					
	6.3 TV					
	6.4 DVD player					
	6.5 Refrigerator					
	6.6 Electric stove					
	6.7 Oven					
	6.8 Microwave					
	6.9 Electric fan					
	6.10 Air conditioner					
	6.11 Cellphone					
	6.12 Tablet					
	6.13 Desktop computer					
	6.14 Laptop					
	6.15 Others, please specify ________________________					
7. Boats	7.1 Own use for fishing					
	7.2 For rent					
	7.3 Others, please specify ________________________					
8. Jewellery						
9. Others, please specify _____________________						

V. Housing

Currently:

(C28) In what type of building/house does the family reside? ……………………………………………………… [ ]

1 - Single house
2 - Duplex
3 - Apartment/accessoria
4 - Commercial/industrial building/house
5 - Condominium/townhouse
99 - Other housing units (e.g., boathouse, treehouse), Please specify ______________________

(C29) What type of construction materials is the roof made of? ………………………………………………… [ ]

1 - Strong materials (galvanized iron, aluminium, tile, concrete, brick, stone, asbestos)
2 - Light materials (cogon, nipa, anahaw)
3 - Salvaged/makeshift materials
4 - Mixed but predominantly strong materials
5 - Mixed but predominantly light materials
6 - Mixed but predominantly salvaged materials
7 - Not Applicable

(C30) What type of construction materials is the outer wall made of? …………………………………………… [ ]

1 - Strong materials (galvanized iron, aluminium, tile, concrete, brick, stone, asbestos)
2 - Light materials (cogon, nipa, anahaw)
3 - Salvaged/makeshift materials
4 - Mixed but predominantly strong materials
5 - Mixed but predominantly light materials
6 - Mixed but predominantly salvaged materials
7 - Not Applicable

(C31) What is the tenure status of the land/lot occupied by your family? ……………………………………….. [ ]

1 - Owned and titled
2 - Owner-like (rights)
3 - Rented
4 - Rent-free without owner's permission (Informal settlers)
5 - Rent-free with owner's permission

If answer is 1 or 2, go to C33. If answer is 3-5, go to C32.

(C32) Who owns the land/lot that is occupied by your family? [ ]

1 - Public entity (like national and local government, GOCCs, etc.)
2 - Private entity

(C33) What is the tenure status of the housing unit occupied by your family? ………………………………… [ ]

1 - Owned
2 - Rented
3 - Rent-free without owner's permission
4 - Rent-free with owner's permission

(C34) How long have your family been living in your current residence? (Number of years) [ ]

(C35) Do you own any other housing unit elsewhere which you also use as residence? (1 - Yes, 0 - No) [ ]

D. FINANCIAL INDICATORS

(D1) Has your household ever taken any loan or credit? (1 - Yes, 0 - No)

(D1.1) If yes, what is the current amount of debt?

(D2) Does your household have a deposit/account or investment in the Philippines? (1 - Yes, 0 - No)

(e.g. savings deposit, mutual funds, time deposit, stock market, etc.)

If YES, got to D2.1 otherwise proceed to D2.2 and D5.

(D2.1) If YES, fill - up the table below.

Accounts	(D2.11) How many accounts do you have?	(D2.12) What is the current interest rate?	(D2.13) Rank 3 of your accounts base on the amount. (1 - being the highest)	(D2.14) Name of bank
1. Savings Account				
2. Current Account				
3. Time Deposit				
4. Savings Certificate				
5. Bond (e.g., Treasury bond/bill)				
6. Mutual Fund				
7. Others, please specify ____________________________				

(D2.2) If NO, what are the reasons why your family does not have an account? Tick all that applies. **[Multiple Answers]**

- 1 - Minimum balance is too high
- 2 - Do not like to deal with banks
- 3 - Service charges are too high
- 4 - Bank/institution's location is far
- 5 - Don't have enough money
- 6 - Don’t need a bank/cash account
- 7 - Don't trust bank/institution
- 8 - Can't manage an account
- 99 - Others, please specify ___________________

For items D3 to D5, tick all that applies. **[Multiple Answers]**

(D3) From your ranking, what are the reasons you chose this bank/institution for your biggest deposit?

- 1 - Proximity to home
- 2 - Proximity to workplace
- 3 - High interest rates
- 4 - Attractive charges for services
- 5 - Efficient service
- 6 - Courteousness of the staff
- 7 - Variety of services, products, instruments offered
- 8 - Internet banking services
- 9 - Personal acquaintances
- 10 - The bank used by employer/or my business
- 11 - It is a major bank
- 12 - Already has another account/loan with bank
- 13 - Bank specified/selected by the source institution of fund
- 14 - No particular reason
- 15 - Don't know/ don't remember
- 99 - Others, please specify ______________________________________

(D4) What are reasons why you are saving your money?

- 1 - House Improvement
- 2 - Medical Expenses
- 3 - Educational expenses
- 4 - For business start-up
- 5 - Business expansion
- 6 - Pay debt
- 7 - Home appliances
- 8 - House & lot purchase
- 9 - Lot purchase
- 10 - House/condo purchase
- 11 - Car loan vehicle
- 99 - Others, please specify _______________________________

(D5) If you have surplus money, where will you put the money?

- 1 - Deposit/Save on Bank
- 2 - Investments (Stocks, Mutual Funds, UITFS)
- 3 - Put up/ Invest in Business/Buy goods for sale/inventory
- 4 - Keep in piggy bank for emergency
- 5 - Pay debt
- 6 - For tuition
- 7 - Life insurance/Pension plan
- 8 - Educational plan
- 9 - Buy car/appliances
- 10 - Buy house/condo
- 11 - Buy land
- 12 - Renovate/house improvements
- 13 - Vacation travel local/abroad
- 14 - Shopping
- 15 - Help parents/relatives
- 16 - Give to charity/church
- 99 - Others, please specify ___________________________

(D6) Do you have contributions in ……………………. (1 - Yes, 0 - No)

1. Philhealth
2. SSS
3. GSIS
4. Pag-ibig Fund
5. Retirement Premiums
6. Life and Accident Insurance
7. Medical Insurance
8. Property Insurance
9. Crop Insurance
10. Others, please specify ________________________

(D7) Please indicate your level of agreement or disagreement to the following Financial Attitudes. **[SHOW CARDS]**

1. I have a habit of saving money regularly
2. I prefer not to think about money
3. I like to know exactly where my money is spent each month
4. I just don't earn enough money to save regularly
5. I don't think I'm saving enough for the future
6. I feel pressured to have the things my friends and neighbors have
7. I would like to save money and increase personal wealth
8. I have the ability to save money and increase personal wealth
9. I have difficulty paying my monthly bills

Codes of Levels of Agreement/Disagreement
1 - Strongly Disagree
2 - Disagree
3 - Neutral
4 - Agree
5 - Strongly Agree

E. ABOUT METROBANK

With respect to Metrobank as an institution, please indicate your level of agreement/disagreement on the following items.
[SHOW CARDS]

(E1) Reliability and Consistency
- [ ] (E1.1) Takes its commitments seriously enough
- [ ] (E1.2) Changes policies with good reason
- [ ] (E1.3) Tries hard to keep its promise

(E2) Integrity and Honesty
- [ ] (E2.1) Takes actions that are consistent in its missions
- [ ] (E2.2) Acknowledges the mistakes it has made
- [ ] (E2.3) Not influenced by politics
- [ ] (E2.4) Takes responsible actions to address an issue or crisis

(E3) Credibility
- [ ] (E3.1) Does not ignore the views of others who disagree with them
- [ ] (E3.2) Partners with NGOs, government, 3rd parties to address societal need

(E4) Fairness
- [ ] (E4.1) Is committed to important process for making decisions
- [ ] (E4.2) Makes a good faith effort to treat everyone even-handedly

(E5) Caring and Concern
- [ ] (E5.1) Can be counted on to do the right thing
- [ ] (E5.2) Listens to customer needs
- [ ] (E5.3) Places customers ahead of profit
- [ ] (E5.3) Creates programs that positively impact the local community

(E6) Competent
- [ ] (E6.1) Has the necessary skills to carry out its jobs
- [ ] (E6.2) Is generally staffed by competent employees
- [ ] (E6.3) Offers high quality products or services
- [ ] (E6.4) Is an innovation of new services or ideas
- [ ] (E6.5) Has highly-regarded and widely armed top leaders
- [ ] (E6.6) Ranks on a global or national top institutions

Codes for Level of Agreement/Disagreement
1 - Strongly disagree
2 - Disagree
3 - Neutral
4 - Agree
5 - Strongly agree

Source: Social Trust and the Management of Risk (pp.100-116)

F. RESPONDENT'S OVERALL PERCEPTION ON MERTOBANK'S SEARCH FOR OUTSTANDING TEACHER APPLICATION

For each statement, indicate the extent of Metrobank Foundation's SOT process. (1 - Not at all, 2 - To some extent, 3 - Significantly)
[SHOW CARDS]

- [ ] (F1) I gained knowledge in my field of expertise
- [ ] (F2) I accessed equipment and expertise not available in our school
- [ ] (F3) I increased my analytical and/or technical skills
- [ ] (F4) I learned techniques for managing and organizing people and projects
- [ ] (F5) I obtained advancements such as promotions, trainings/scholarships, etc.
- [ ] (F6) I increased my ability to influence and to make changes in our work
- [ ] (F7) I apply my skills and knowledge gained during the award in our work
- [ ] (F8) I introduced new practices or innovations in our organization as a result of skills/knowledge acquired through the award

SOT Survey Questionnaire 2 (For the School Head only)
Strictly Confidential

Control No.: A P - _ _ _

Appendix B: Supplementary file - Questionnaire Survey Data of Finalists in the Metrobank Foundation Search for Outstanding Teachers 1988-2010

Majah-Leah V. Ravago[1], Claire Dennis S. Mapa[2]
1. Ateneo de Manila University 2. University of the Philippines

ENUMERATOR'S INFORMATION
Name of Enumerator: ______________________________

Date:
Time Started:
Time Finished:

Note for the Enumerator:
____
____

For multiple finalists/winners

No.	Name of Applicant	1- Finalist, 2 - Winner	Year
1			
2			
3			
4			
5			

***use another sheet if necessary**

A. SCHOOL AFFILIATION

(A1) Name of School Head: ______________________________
(A1.1) Surname (A1.2) Given Name (A1.3) Middle Initial (A1.4) Ext.(e.g. Sr. Jr)

(A2) School Name: ______________________________

(A3) School Address: ______________________________
(A3.1) Barangay (A3.2) Municipality/City (A3.3) Province (A3.4) Region

(A4) Type of School: 1 - Public 2 - Private

(A5) Location of School: 1 - Rural 2 - Central 3 - Other Urban

(A6) Student Population:

(A7) Do you know the SOT applicant during the time of his/her application? (1 - Yes, 0 - No)
(A7.1) If YES in A7, in what capacity did you know the SOT applicant from the time of application?
(A7.11) As a fellow teacher
(A7.12) As a supervisor
(A7.13) As a colleague from another group/organization/institution
A7.14) Other, please specify ______________

(A7.2) If NO in A7, did you know the applicant after the SOT? (1 - Yes, 0 - No)
If Yes in A7.2, in what year did you know the applicant?

(A7.3) If NO in A7.2, were you aware that there was an SOT applicant in your school? (1 - Yes, 0 - No)

Warning!: If the answer in A7, A7.2 and A7.3 is NO, do not proceed, otherwise seek for other Key Informant (of higher position) who knows the applicant/s and is capable of giving information about the school.

(A8) Teachers' Educational Profile

Number of faculty members with the highest educational attainment	(A8.1) during the first SOT applicant from your school	(A8.2) currently
1. Bachelor's Degree		
2. Master's Degree		
3. Doctorate's Degree		
4. Total		

(A9) Performance Evaluation/Rating of SOT Applicant

(A9.1) Year	(A9.2) Rating*

Note: Rating refers to the Supervisor's rating (primary & secondary) or students rating whichever is applicable
Use other sheet for another applicant [For multiple winners/finalists]

B. QUESTIONS RELATED TO SCHOOL

(B1) What is the average ……..	(B1.1) during the first SOT applicant in your school?	(B1.2) currently?
1. Number of Enrolees		
2. Number of Graduates		
3. Number of Drop-outs		
4. Retention Rate		
5. Completion Rate		
6. Survival Rate		

School Physical Characteristic

(B2) How many do you have ……..	(B2.1) during the first SOT applicant in your school?	(B2.2) currently?	(B2.3) Do you thin applicant directly c contribute to this cha school? (1 - Yes, 0 -
1. Classrooms			
2. Libraries			
3. Laboratories			
4. Computers exclusive for teaching purposes			
5. Canteens			
6. Dorms			
7. Sport facilities (e.g. gym, basketball court)			
8. Others, please specify ___________________________			

(B3) Does your school solicit funds from the ………..	(B3.1) during the first SOT applicant in your school?		(B3.2) currently?		(B3.3) Is having ar positively contribu school ability to rais for the scho (1 - Yes, 0 -
	(1 - Yes, 0 - No)	(B3.11) If YES, specify the amount	(1 - Yes, 0 - No)	(B3.21) If YES, specify the amount	
1. Government					
2. Private Sector					
3. Others sources					

C. QUESTIONS RELATED TO FELLOW TEACHERS

(C1) Please rate your fellow teachers (overall) according to …………………	(C1.1) during one of your colleague became a finalist/winner in SOT?	(C1.2) currently?	(C1.3) D havin applicar contribute all the tea sc (1 - Y
1. Values (especially love of country and pride as a Filipino)			
2. General awareness of socio-economic and political issues			
3. General attitude towards the profession, students, peers and school administrators			
4. Civic and community involvement			
5. Personal Discipline and personal/family life			
6. Specialization and/or major subject taught			
7. Communication skills and personality			

Ratings: 10 - Highest and 1 - Lowes

(C2) Was there any succeeding applicants in SOT coming from your school after the last finalist [NAME]*? (1 - Yes, 0 - No)
Refer to page 1 of the questionnaire.

(C2.1) If YES, how many applicants?

(C2.2) Where did the succeeding applicants get the information about SOT?

1 - Referral from previous applicant/s from this institution
3 - Referral from previous applicant/s from other schools
3 - Endorsement by the Head of this school
4 - Endorsement by the Head of other school
5 - Referral from school Supervisors, Superintendent, etc.
6 - Social Media
7 - Media
8 - Others, please specify _______________________

D. QUESTIONS RELATED TO STUDENTS

(D1) Applicable for Primary and Secondary Schools

No.	(D1.1) Year	Mean Percentage Score on National Achievement Test											
		(D1.2) National Elementary Achievement Test						(D1.3) National Secondary Achievement Test					
		(D1.21) Math	(D1.22) Science	(D1.23) English	(D1.24) HeKaSi	(D1.25) Filipino	**(D1.26) Overall**	(D1.31) Math	(D1.32) Science	(D1.33) English	(D1.34) Araling Panlipunan	(D1.35) Filipino	**(D1.36) Overall**
1													
2													
3													
4													
5													
6													
7													
8													
9													
10													
11													
12													
13													
14													
15													

Note: Overall rating is the average rating of all the subjects

Note: Consecutive years starting from the latest.

(D2) Applicable for Tertiary Schools only

No.	(D2.1) Year	Mean Percentage Score on Licensure Examination								
		(D2.2) Licensure Examination for …								
		(D2.21) Nursing	(D2.22) Engineering	(D2.23) Chemistry	(D2.24) Education (Teachers)	(D2.25) Accounting	(D2.26) Agriculture & Forestry	(D2.27) Others, please specify		
								A) _______	B) _______	C) _______
1										
2										
3										
4										
5										
6										
7										
8										
9										
10										
11										
12										
13										
14										
15										

Note: Consecutive years starting from the latest.

E. FINANCIAL INDICATORS

(E1) Do you or any member of the household have a deposit/account or investment in the Philippines? (1 - Yes, 0 - No) ☐

(e.g. savings deposit, mutual funds, time deposit, stock market, etc.)

If YES, got to E1.1 otherwise proceed to E1.2 and E4.

(E2.1) If YES, fill - up the table below.

Accounts	(E1.11) How many accounts do you have?	(E1.12) What is the current interest rate?	(E1.13) Rank 3 of your accounts base on the amount. (1 - being the highest)	(E1.14) Name of bank
1. Savings Account				
2. Current Account				
3. Time Deposit				
4. Savings Certificate				
5. Bond				
6. Mutual Fund				
7. Others, please specify ___________________________				

(E1.2) If NO, what are the reasons why your family does not have an account? Tick all that applies. **[Multiple Answers]**

- ☐ 1 - Minimum balance is too high
- ☐ 2 - Do not like to deal with banks
- ☐ 3 - Service charges are too high
- ☐ 4 - Bank/institution's location is far
- ☐ 5 - Don't have enough money
- ☐ 6 - Don’t need a bank/cash account
- ☐ 7 - Don't trust bank/institution
- ☐ 8 - Can't manage an account
- ☐ 99 - Others, please specify ___________________

For items E2 to E4, tick all that applies. **[Multiple Answers]**

(E2) From your ranking, what are the reasons you chose this bank/institution for your biggest deposit?

- ☐ 1 - Proximity to home
- ☐ 2 - Proximity to workplace
- ☐ 3 - High interest rates
- ☐ 4 - Attractive charges for services
- ☐ 5 - Efficient service
- ☐ 6 - Courteousness of the staff
- ☐ 7 - Variety of services, products, instruments offered
- ☐ 8 - Internet banking services
- ☐ 9 - Personal acquaintances
- ☐ 10 - The bank used by employer/or my business
- ☐ 11 - It is a major bank
- ☐ 12 - Already has another account/loan with bank
- ☐ 13 - Bank specified/selected by the source institution of fund
- ☐ 14 - No particular reason
- ☐ 15 - Don't know/ don't remember
- ☐ 99 - Others, please specify _____________________________________

(E3) What are reasons why you are saving your money?

- ☐ 1 - House Improvement
- ☐ 2 - Medical Expenses
- ☐ 3 - Educational expenses
- ☐ 4 - For business start-up
- ☐ 5 - Business expansion
- ☐ 6 - Pay debt
- ☐ 7 - Home appliances
- ☐ 8 - House & lot purchase
- ☐ 9 - Lot purchase
- ☐ 10 - House/condo purchase
- ☐ 11 - Car loan vehicle
- ☐ 99 - Others, please specify ______________________________

(E4) If you have surplus money, where will you put the money?

- ☐ 1 - Deposit/Save on Bank
- ☐ 2 - Investments (Stocks, Mutual Funds, UITFS)
- ☐ 3 - Put up/ Invest in Business/Buy goods for sale/inventory
- ☐ 4 - Keep in piggy bank for emergency
- ☐ 5 - Pay debt
- ☐ 6 - For tuition
- ☐ 7 - Life insurance/Pension plan
- ☐ 8 - Educational plan
- ☐ 9 - Buy car/appliances
- ☐ 10 - Buy house/condo
- ☐ 11 - Buy land
- ☐ 12 - Renovate/house improvements
- ☐ 13 - Vacation travel local/abroad
- ☐ 14 - Shopping
- ☐ 15 - Help parents/relatives
- ☐ 16 - Give to charity/church
- ☐ 99 - Others, please specify __________________________

(E5) Do you have contributions in ……………………. (1 - Yes, 0 - No)

1. Philhealth ☐
2. SSS ☐
3. GSIS ☐
4. Pag-ibig Fund ☐
5. Retirement Premiums ☐
6. Life and Accident Insurance ☐
7. Medical Insurance ☐
8. Property Insurance ☐
9. Crop Insurance ☐
10. Others, please specify _______________________ ☐

(E6) Please indicate your level of agreement or disagreement to the following Financial Attitudes. **[SHOW CARDS]**

1. I have a habit of saving money regularly ☐
2. I prefer not to think about money ☐
3. I like to know exactly where my money is spent each month ☐
4. I just don't earn enough money to save regularly ☐
5. I don't think I'm saving enough for the future ☐
6. I feel pressured to have the things my friends and neighbors have ☐
7. I would like to save money and increase personal wealth ☐
8. I have the ability to save money and increase personal wealth ☐
9. I have difficulty paying my monthly bills ☐

Codes of Levels of Agreement/Disagreement
1 - Strongly Disagree
2 - Disagree
3 - Neutral
4 - Agree
5 - Strongly Agree

F. ABOUT METROBANK

With respect to Metrobank as an institution, please indicate your level of agreement/disagreement on the following items.
[SHOW CARDS]

(F1) Reliability and Consistency

- ☐ (F1.1) Takes its commitments seriously enough
- ☐ (F1.2) Changes policies with good reason
- ☐ (F1.3) Tries hard to keep its promise

(F2) Integrity and Honesty

- ☐ (F2.1) Takes actions that are consistent in its missions
- ☐ (F2.2) Acknowledges the mistakes it has made
- ☐ (F2.3) Not influenced by politics
- ☐ (F2.4) Takes responsible actions to address an issue or crisis

(F3) Credibility

- ☐ (F3.1) Does not ignore the views of others who disagree with them
- ☐ (F3.2) Partners with NGOs, government, 3rd parties to address societal need

(F4) Fairness

- ☐ (F4.1) Is committed to important process for making decisions
- ☐ (F4.2) Makes a good faith effort to treat everyone even-handedly

(F5) Caring and Concern

- ☐ (F5.1) Can be counted on to do the right thing
- ☐ (F5.2) Listens to customer needs
- ☐ (F5.3) Places customers ahead of profit
- ☐ (F5.3) Creates programs that positively impact the local community

(F6) Competent

- ☐ (F6.1) Has the necessary skills to carry out its jobs
- ☐ (F6.2) Is generally staffed by competent employees
- ☐ (F6.3) Offers high quality products or services
- ☐ (F6.4) Is an innovation of new services or ideas
- ☐ (F6.5) Has highly-regarded and widely armed top leaders
- ☐ (F6.6) Ranks on a global or national top institutions

Codes for Level of Agreement/Disagreement
1 - Strongly disagree
2 - Disagree
3 - Neutral
4 - Agree
5 - Strongly agree

Source: Social Trust and the Management of Risk (pp.100-116)
