# Supplementary material for: Survey data of finalists and winners in the search for outstanding teachers in the Philippines, 1988–2010
Source: Data Brief. 2020 Aug 27;32:106238. doi: 10.1016/j.dib.2020.106238 (PMC7474403; doi:10.1016/j.dib.2020.106238)
Supplement: Supplementary file 3 — Appendix C. Operational definitions of the specific terms used in the survey questionnaire (with this article) [file mmc3.docx]

**Annex C. Concepts and definitions**

Most of the concepts and definitions used in the survey questionnaire, follow the standard definitions used in Family Income and Expenditure Survey [2]. Here, we define only those terms that are not used in official household surveys.

1. **Teacher questionnaire**

**Block B - Teacher’s competence**

- Load – this refers to the total number of hours per week spent by the teacher in a particular activity related to his or her profession
- Advisee – this refers to an undergraduate and graduate student being handled / guided by a professor in his or her thesis/ dissertation.
- Local – this area covers barangay, city/ municipality, province and region
- Short term – refers to a duration of 1 day to 3 weeks
- Long Term – refers to a duration of more than 3 weeks
- Position (B9) – this refers to any non-political position held by the teacher in a particular agency
- Position (B10) – this refers to any local / national political position held by the teacher

**Block C - General information on household**

- Current Value – refers to the total number of assets including the items bought during application if these assets are still available.
- Housing – questions from C28 to C35 uses the teacher’s present/ current residence as the main reference

**Block D - Financial indicators**

- Deposit/ Account/ Investment – this refer to any of the following: savings deposit, mutual funds, time deposit, stock market, etc.

1. **School head questionnaire**

**Block A - School affiliation**

Rating – this refers to the Supervisor’s rating (primary and secondary) or students rating whichever is applicable.

**Block B - Questions related to school**

- No. of Drop-outs – total number of students who do not finish a particular grade / year level. It does not capture pupils/ students who finish a grade/ year level but do not enroll in the next grade/ year level the following school year.
- Retention rate – determines the degree of pupils/ students in a school year who continue to be in school in the succeeding year.
- Completion rate – measures the percentage of grade/ year 1 entrants who graduate in elementary/ secondary education. It is available only up to the division level and above.
- Survival rate – computes the percentage of a cohort of pupils/ students who are able to reach grade VI/ Year IV. it is used to assess the internal efficiency and “wastage” in education.

**Block C - Questions related to fellow teachers**

SOT – Search for Outstanding Teachers

**Block E - Financial indicators**

- Deposit/ Account/ Investment – this refer to any of the following: savings deposit, mutual funds, time deposit, stock market, etc.
